# 2-(1*H*-Pyrazol-3-yl)pyridinium chloride monohydrate

**DOI:** 10.1107/S1600536809028402

**Published:** 2009-07-25

**Authors:** Tatiana R. Amarante, Isabel S. Gonçalves, Filipe A. Almeida Paz

**Affiliations:** a Department of Chemistry, University of Aveiro, CICECO, 3810-193 Aveiro, Portugal

## Abstract

The title organic salt, C_8_H_8_N_3_^+^·Cl^−^·H_2_O, exhibits a rich hydrogen-bonding network involving all constituent species. The water mol­ecules are engaged in strong O—H⋯Cl inter­actions with the chloride anions, two neighboring protonated 2-(1*H*-pyrazol-3-yl)pyridinium species inter­act *via* N—H⋯N bonds with two pyrazole rings. Further, a short and highly directional C—H⋯O inter­action is observed connecting the pyridinium ring to the water mol­ecule of crystallization. Weak C—H⋯Cl and N—H⋯Cl inter­actions contribute to the stabilization of the crystal structure.

## Related literature

For related structures with 2-(3-pyrazol­yl)pyridine or its derivatives, see: Coelho *et al.* (2006 ▶, 2007 ▶); Fleming *et al.* (1998 ▶); Jones *et al.* (1997 ▶); Lam *et al.* (1997 ▶); Leita *et al.* (2004 ▶); Li (2007 ▶); Liu *et al.* (2006 ▶); Mokuolu *et al.* (2007 ▶); Hu, Li *et al.* (2006 ▶); Hu, Wang *et al.* (2006 ▶); Hu *et al.* (2008 ▶); Huo *et al.* (2006 ▶); Ward, Fleming *et al.* (1998 ▶); Ward, Mann *et al.* (1998 ▶). For detailed background to the role of hydrogen bonds in the supra­molecular organization of organic crystals, see: Nangia & Desiraju (1998 ▶). For general background studies on crystal engineering approaches from our research group, see: Paz & Klinowski (2003 ▶); Paz *et al.* (2002 ▶). For a description of the graph-set notation for hydrogen-bonded aggregates, see: Bernstein *et al.* (1995 ▶). For a description of the Cambridge Structural Database, see: Allen (2002 ▶).
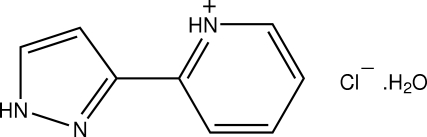


## Experimental

### 

#### Crystal data

C_8_H_8_N_3_^+^·Cl^−^·H_2_O
*M* *_r_* = 199.64
Triclinic, 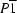

*a* = 6.8487 (2) Å
*b* = 8.3523 (3) Å
*c* = 9.0843 (3) Å
α = 114.693 (1)°
β = 99.867 (2)°
γ = 91.097 (2)°
*V* = 462.75 (3) Å^3^
*Z* = 2
Mo *K*α radiation
μ = 0.38 mm^−1^
*T* = 150 K
0.18 × 0.15 × 0.09 mm

#### Data collection

Bruker X8 Kappa CCD APEXII diffractometer
Absorption correction: multi-scan (*SADABS*; Sheldrick, 1997 ▶) *T*_min_ = 0.930, *T*_max_ = 0.951
26245 measured reflections
4422 independent reflections
3687 reflections with *I* > 2σ(*I*)
*R*_int_ = 0.024

#### Refinement

*R*[*F* ^2^ > 2σ(*F* ^2^)] = 0.048
*wR*(*F* ^2^) = 0.162
*S* = 1.07
4422 reflections
124 parameters
3 restraints
H atoms treated by a mixture of independent and constrained refinement
Δρ_max_ = 1.26 e Å^−3^
Δρ_min_ = −0.99 e Å^−3^

### 

Data collection: *APEX2* (Bruker, 2006 ▶); cell refinement: *SAINT-Plus* (Bruker, 2005 ▶); data reduction: *SAINT-Plus*; program(s) used to solve structure: *SHELXTL* (Sheldrick, 2008 ▶); program(s) used to refine structure: *SHELXTL*; molecular graphics: *DIAMOND* (Brandenburg, 2009 ▶); software used to prepare material for publication: *SHELXTL*.

## Supplementary Material

Crystal structure: contains datablocks global, I. DOI: 10.1107/S1600536809028402/tk2502sup1.cif

Structure factors: contains datablocks I. DOI: 10.1107/S1600536809028402/tk2502Isup2.hkl

Additional supplementary materials:  crystallographic information; 3D view; checkCIF report

**Fig. 4.**
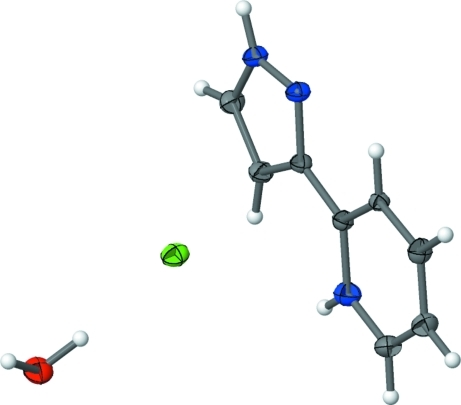
Asymmetric unit of the title compound will all non-hydrogen atoms represented as thermal ellipsoids drawn at the 50% probability level.

Enhanced figure: interactive version of Fig. 4

**Fig. 5.**
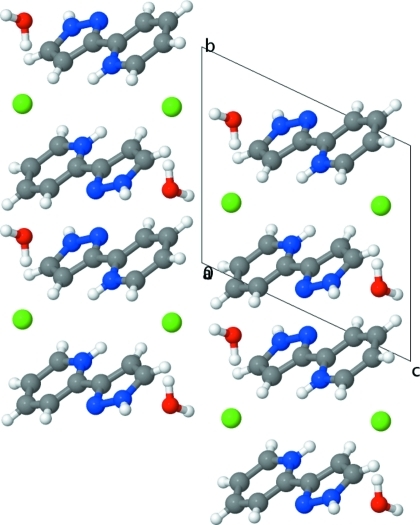
Crystal packing of the title compound.

Enhanced figure: interactive version of Fig. 5

## Figures and Tables

**Table 1:** Hydrogen-bond geometry (Å, °)

*D*—H⋯*A*	*D*—H	H⋯*A*	*D*⋯*A*	*D*—H⋯*A*
N1—H1⋯N2^i^	0.88	2.25	2.9502 (16)	137
O1*W*—H1*A*⋯Cl1^ii^	0.940 (19)	2.176 (10)	3.1113 (13)	173 (2)
O1*W*—H1*B*⋯Cl1	0.95 (2)	2.279 (11)	3.2106 (12)	170 (2)
C5—H5⋯O1*W*^iii^	0.95		2.7203 (16)	156
C8—H8⋯Cl1^iv^	0.95		3.5862 (18)	137
N3—H3⋯Cl1^v^	0.88	2.94	3.7915 (15)	162
C2—H2⋯Cl1^v^	0.95		3.5604 (13)	159
C6—H6⋯Cl1^vi^	0.95		3.5329 (14)	127
C7—H7⋯Cl1^vi^	0.95		3.5649 (14)	123

Symmetry codes: (i) 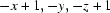; (ii) 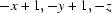; (iii) 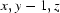; (iv) 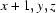; (v) 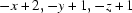; (vi) 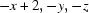.

**Fig. 1.**
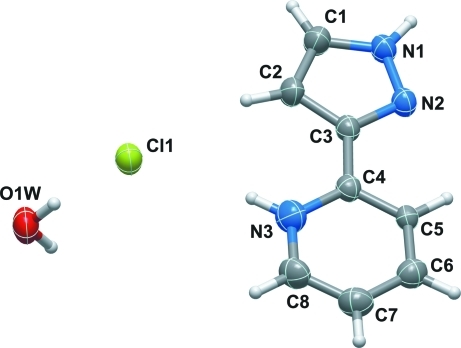
Asymmetric unit of (I) with all non-hydrogen atoms represented as thermal displacement ellipsoids drawn at the 80% probability level and hydrogen atoms as small spheres with arbitrary radii. The labeling scheme is provided for all non-hydrogen atoms.

**Fig. 2.**
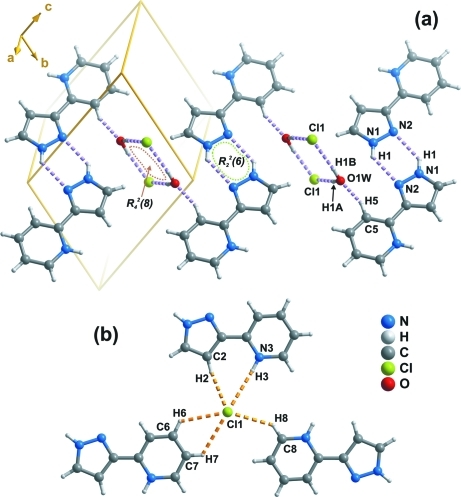
Hydrogen bonds interconnecting the chemical moieties present in (I). (a) Strong N—H···N, O—H···Cl and short C—H···O interactions (violet dashed lines), leading to the formation of a 1-D supramolecular tape composed by the alternation of R42(8), R22(6) and S graph set motifs. (b) Weak N—H···Cl and C—H···Cl interactions (orange dashed lines). For clarity all symmetry transformations used to generate equivalent atoms have been omitted. See Table 1 for geometric details of the highlighted hydrogen bonding interactions.

**Fig. 3.**
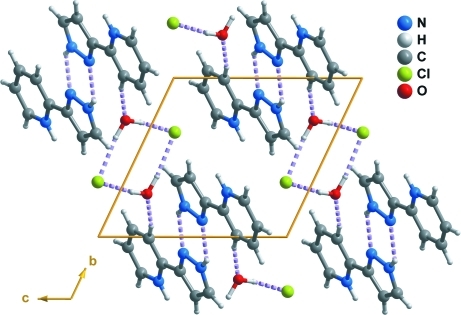
Crystal packing viewed along the [100] direction of the unit cell. Strong hydrogen bonds (N—H···N, O—H···Cl and short C—H···O) are represented as violet dashed lines.

## supplementary crystallographic information

### Comment

In recent years, molecules derived from 2-(3-pyrazolyl)pyridine have been very much explored in coordination chemistry as *N,N*-chelating moieties, *N,N*-bridging (the two N-atoms from the pyrazolyl aromatic ring), or even a combination of these two coordination modes, in conjunction with other ligands to produce functional complexes which, ultimately, may find applications in catalysis or as photoluminescent devices. Indeed, a search in the literature and in the Cambridge Structural Database (CSD, Version of November 2008 with three updates; Allen, 2002) reveals a plethora of transition metal complexes: Ag^+^ (Li, 2007), Cd^2+^ (Hu *et al.*, 2008; Liu *et al.*, 2006; Hu, Wang *et al.*, 2006; Huo *et al.*, 2006), Cu^2+^ (Hu, Li *et al.*, 2006; Fleming *et al.*, 1998; Mokuolu *et al.*, 2007), Cu^+^ (Lam *et al.*, 1997), Fe^2+^ (Leita *et al.*, 2004), Fe^3+^ (Jones *et al.*, 1997), In^3+^ (Ward, Mann *et al.*, 1998), Pd^2+^ (Ward, Fleming *et al.*,  1998), Ru^2+^ (Lam *et al.*, 1997), Zn^2+^ (Hu *et al.*, 2008).

Our research group has been particularly interested in the use of this molecule and its derivatives. For example, we have reported the crystal structures of both the molybdenum complex *cis*-[Mo(CO)_4_*L*] (Coelho *et al.*, 2006) and the organic ligand *L* {ethyl[3-(2-pyridyl)-1-pyrazolyl]acetate} (Coelho *et al.*, 2007). We have recently isolated single crystals of (I) as a secondary (minor) phase (see Experimental). Following our on-going interest in the structural details of organic crystals (Paz & Klinowski, 2003; Paz *et al.*, 2002), here we report the supramolecular structure at 150 K of the monohydrate form of the title salt: C_8_H_8_N_3_^+^Cl^-.^H_2_O, (I).

The asymmetric unit of the title compound, (I), is composed of a cationic C_8_H_8_N_3_^+^ moiety (protonated at the pyridine ring), one chloride anion and one water molecule of crystallization (Fig. 1). The two aromatic rings of 2-(3-pyrazolyl)pyridinium can be considered as coplanar (the average planes containing the rings are tilted by only *ca* 1°).

The existence of several chemical groups capable of hydrogen bonding either as donors or acceptors leads to the formation of a complex supramolecular network. Water molecules and chloride anions are involved in strong (*d*_D···A_ ranging between *ca* 3.11 and 3.21 Å) and highly directional [<(DHA) angles above 170° - see Table 1] O—H···Cl hydrogen bonding interactions forming a *R_2_^2^(8)* graph set motif as depicted in Fig. 2a (Bernstein *et al.*, 1995). It is important to emphasize that the water molecule itself acts as the acceptor in an unusual C—H···O_water_ interaction. Indeed, even though this interaction is not considered as classic (Nangia & Desiraju, 1998), in the structure of (I) the *d*_D···A_ distance is considered short [2.7203 (16) Å] and the <(DHA) angle is 156°. Thus, these geometric parameters allow us to infer that this interaction seems to play an important role in the supramolecular organization of (I). In addition, the close proximity of the pyrazolyl rings belonging to two adjacent 2-(3-pyrazolyl)pyridinium moieties promote the formation of two N—H···N interactions describing a *R_2_^2^(6)* motif (Fig. 2a). The alternation between the two aforementioned graph set motifs and the single C—H···O_water_ interaction leads to the formation of a supramolecular tape (solely based on strong interactions) running parallel to the [011] vector of the unit cell (Fig. 3).

The crystal packing of (I) is further promoted by the existence of a number of weak C—H···Cl and one N—H···Cl hydrogen bonding interactions as shown in Fig. 2b (Table 1), which establish connections between adjacent supramolecular tapes (not shown).

### Experimental

Crystals of (I) were isolated as a secondary product while reacting in dichloromethane MoO_2_Cl_2_ with 2-(3-pyrazolyl)pyridine.

### Refinement

Hydrogen atoms bound to carbon and nitrogen were located at their idealized positions and were included in the final structural model in riding model approximation with C—H = 0.95 Å and N—H = 0.88 Å, and with *U*(H) set to 1.2*U*_eq_(C, N).

H atoms associated with the water molecule of crystallization were directly located from difference Fourier maps and included in the structure with the O—H and H···H distances restrained to 0.95 (1) and 1.55 (1) Å, respectively, with *U*(H) set to 1.5*U*_eq_(O).

The final difference Fourier map synthesis showed the highest peak (1.26 eÅ^-3^) located at 0.25 Å from the C5 atom.

### Crystal data

**Table:**

C_8_H_8_N_3_^+^·Cl^−^·H_2_O	*Z* = 2
*M**_r_* = 199.64	*F*(000) = 208
Triclinic, *P*1	*D*_x_ = 1.433 Mg m^−^^3^
Hall symbol: -P 1	Mo *K*α radiation, λ = 0.71073 Å
*a* = 6.8487 (2) Å	Cell parameters from 9894 reflections
*b* = 8.3523 (3) Å	θ = 2.5–39.6°
*c* = 9.0843 (3) Å	µ = 0.38 mm^−^^1^
α = 114.693 (1)°	*T* = 150 K
β = 99.867 (2)°	Prism, colourless
γ = 91.097 (2)°	0.18 × 0.15 × 0.09 mm
*V* = 462.75 (3) Å^3^	

### Data collection

**Table:**

Bruker X8 Kappa CCD APEXII diffractometer	4422 independent reflections
Radiation source: fine-focus sealed tube	3687 reflections with *I* > 2σ(*I*)
graphite	*R*_int_ = 0.024
ω and φ scans	θ_max_ = 36.3°, θ_min_ = 3.5°
Absorption correction: multi-scan (*SADABS*; Sheldrick, 1997)	*h* = −11→11
*T*_min_ = 0.930, *T*_max_ = 0.951	*k* = −13→13
26245 measured reflections	*l* = −15→15

### Refinement

**Table:**

Refinement on *F*^2^	Primary atom site location: structure-invariant direct methods
Least-squares matrix: full	Secondary atom site location: difference Fourier map
*R*[*F*^2^ > 2σ(*F*^2^)] = 0.048	Hydrogen site location: inferred from neighbouring sites
*wR*(*F*^2^) = 0.162	H atoms treated by a mixture of independent and constrained refinement
*S* = 1.07	*w* = 1/[σ^2^(*F*_o_^2^) + (0.0872*P*)^2^ + 0.3244*P*] where *P* = (*F*_o_^2^ + 2*F*_c_^2^)/3
4422 reflections	(Δ/σ)_max_ = 0.001
124 parameters	Δρ_max_ = 1.26 e Å^−^^3^
3 restraints	Δρ_min_ = −0.99 e Å^−^^3^

### Special details

**Table:**

Geometry. All e.s.d.'s (except the e.s.d. in the dihedral angle between two l.s. planes) are estimated using the full covariance matrix. The cell e.s.d.'s are taken into account individually in the estimation of e.s.d.'s in distances, angles and torsion angles; correlations between e.s.d.'s in cell parameters are only used when they are defined by crystal symmetry. An approximate (isotropic) treatment of cell e.s.d.'s is used for estimating e.s.d.'s involving l.s. planes.
Refinement. Refinement of *F*^2^ against ALL reflections. The weighted *R*-factor *wR* and goodness of fit *S* are based on *F*^2^, conventional *R*-factors *R* are based on *F*, with *F* set to zero for negative *F*^2^. The threshold expression of *F*^2^ > σ(*F*^2^) is used only for calculating *R*-factors(gt) *etc*. and is not relevant to the choice of reflections for refinement. *R*-factors based on *F*^2^ are statistically about twice as large as those based on *F*, and *R*- factors based on ALL data will be even larger.

### Fractional atomic coordinates and isotropic or equivalent isotropic displacement parameters (Å^2^)

**Table:**

	*x*	*y*	*z*	*U*_iso_*/*U*_eq_	
N1	0.61184 (17)	0.19217 (15)	0.62050 (15)	0.0183 (2)	
H1	0.5010	0.1500	0.6343	0.022*	
N2	0.70080 (17)	0.10464 (15)	0.49209 (14)	0.0173 (2)	
N3	1.1678 (2)	0.28024 (18)	0.43052 (18)	0.0245 (2)	
H3	1.1939	0.3832	0.5176	0.029*	
C1	0.7105 (2)	0.35120 (18)	0.72575 (18)	0.0209 (2)	
H1C	0.6738	0.4335	0.8239	0.025*	
C2	0.8746 (2)	0.37168 (17)	0.66403 (18)	0.0195 (2)	
H2	0.9742	0.4694	0.7095	0.023*	
C3	0.86126 (18)	0.21518 (16)	0.51834 (16)	0.0153 (2)	
C4	0.99968 (18)	0.16869 (16)	0.40505 (16)	0.0155 (2)	
C5	0.96578 (17)	0.01058 (15)	0.27103 (14)	0.01290 (18)	
H5	0.8509	−0.0658	0.2533	0.015*	
C6	1.0876 (2)	−0.04322 (19)	0.16188 (17)	0.0206 (2)	
H6	1.0589	−0.1564	0.0705	0.025*	
C7	1.2539 (2)	0.0651 (2)	0.18193 (19)	0.0231 (3)	
H7	1.3393	0.0285	0.1042	0.028*	
C8	1.2942 (2)	0.2287 (2)	0.31810 (19)	0.0220 (2)	
H8	1.4081	0.3052	0.3343	0.026*	
Cl1	0.67400 (5)	0.34331 (4)	0.14179 (4)	0.02184 (10)	
O1W	0.68083 (18)	0.73153 (15)	0.15022 (15)	0.0261 (2)	
H1A	0.575 (3)	0.719 (3)	0.064 (2)	0.039*	
H1B	0.692 (4)	0.623 (2)	0.159 (3)	0.039*	

### Atomic displacement parameters (Å^2^)

**Table:**

	*U*^11^	*U*^22^	*U*^33^	*U*^12^	*U*^13^	*U*^23^
N1	0.0160 (4)	0.0180 (5)	0.0194 (5)	−0.0016 (4)	0.0048 (4)	0.0061 (4)
N2	0.0165 (4)	0.0166 (4)	0.0173 (4)	−0.0016 (3)	0.0037 (4)	0.0059 (4)
N3	0.0234 (5)	0.0216 (5)	0.0292 (6)	−0.0003 (4)	0.0076 (5)	0.0107 (5)
C1	0.0206 (6)	0.0170 (5)	0.0217 (6)	0.0001 (4)	0.0062 (5)	0.0042 (4)
C2	0.0182 (5)	0.0147 (5)	0.0228 (6)	−0.0012 (4)	0.0049 (4)	0.0051 (4)
C3	0.0144 (5)	0.0142 (4)	0.0172 (5)	−0.0002 (4)	0.0023 (4)	0.0068 (4)
C4	0.0150 (5)	0.0147 (5)	0.0174 (5)	0.0004 (4)	0.0025 (4)	0.0076 (4)
C5	0.0123 (4)	0.0127 (4)	0.0128 (4)	−0.0003 (3)	0.0011 (3)	0.0052 (3)
C6	0.0219 (6)	0.0208 (5)	0.0184 (5)	0.0033 (4)	0.0051 (4)	0.0073 (4)
C7	0.0219 (6)	0.0259 (6)	0.0249 (6)	0.0042 (5)	0.0096 (5)	0.0123 (5)
C8	0.0190 (5)	0.0227 (6)	0.0276 (6)	0.0003 (4)	0.0077 (5)	0.0129 (5)
Cl1	0.01974 (15)	0.02010 (15)	0.02130 (16)	−0.00223 (11)	0.00433 (11)	0.00468 (11)
O1W	0.0240 (5)	0.0202 (5)	0.0275 (5)	−0.0047 (4)	−0.0012 (4)	0.0065 (4)

### Geometric parameters (Å, °)

**Table:**

N1—N2	1.3471 (16)	C3—C4	1.4572 (18)
N1—C1	1.3485 (18)	C4—C5	1.3521 (17)
N1—H1	0.8800	C5—C6	1.3439 (18)
N2—C3	1.3433 (16)	C5—H5	0.9500
N3—C8	1.389 (2)	C6—C7	1.379 (2)
N3—C4	1.3908 (18)	C6—H6	0.9500
N3—H3	0.8800	C7—C8	1.389 (2)
C1—C2	1.3765 (19)	C7—H7	0.9500
C1—H1C	0.9500	C8—H8	0.9500
C2—C3	1.4061 (18)	O1W—H1A	0.940 (19)
C2—H2	0.9500	O1W—H1B	0.95 (2)
			
N2—N1—C1	112.87 (11)	C5—C4—N3	118.43 (12)
N2—N1—H1	123.6	C5—C4—C3	119.14 (11)
C1—N1—H1	123.6	N3—C4—C3	122.42 (12)
C3—N2—N1	104.03 (10)	C6—C5—C4	122.82 (12)
C8—N3—C4	119.79 (13)	C6—C5—H5	118.6
C8—N3—H3	120.1	C4—C5—H5	118.6
C4—N3—H3	120.1	C5—C6—C7	120.30 (13)
N1—C1—C2	107.03 (12)	C5—C6—H6	119.8
N1—C1—H1C	126.5	C7—C6—H6	119.8
C2—C1—H1C	126.5	C6—C7—C8	118.69 (13)
C1—C2—C3	104.32 (11)	C6—C7—H7	120.7
C1—C2—H2	127.8	C8—C7—H7	120.7
C3—C2—H2	127.8	N3—C8—C7	119.94 (13)
N2—C3—C2	111.76 (11)	N3—C8—H8	120.0
N2—C3—C4	121.41 (11)	C7—C8—H8	120.0
C2—C3—C4	126.84 (11)	H1A—O1W—H1B	110.2 (14)
			
C1—N1—N2—C3	−0.48 (15)	C2—C3—C4—C5	179.05 (13)
N2—N1—C1—C2	0.23 (17)	N2—C3—C4—N3	−179.37 (12)
N1—C1—C2—C3	0.12 (16)	C2—C3—C4—N3	−0.1 (2)
N1—N2—C3—C2	0.55 (15)	N3—C4—C5—C6	0.11 (19)
N1—N2—C3—C4	179.90 (11)	C3—C4—C5—C6	−179.10 (12)
C1—C2—C3—N2	−0.43 (16)	C4—C5—C6—C7	−1.1 (2)
C1—C2—C3—C4	−179.74 (13)	C5—C6—C7—C8	1.0 (2)
C8—N3—C4—C5	0.9 (2)	C4—N3—C8—C7	−0.9 (2)
C8—N3—C4—C3	−179.96 (13)	C6—C7—C8—N3	−0.1 (2)
N2—C3—C4—C5	−0.20 (19)		

### Hydrogen-bond geometry (Å, °)

**Table:**

*D*—H···*A*	*D*—H	H···*A*	*D*···*A*	*D*—H···*A*
N1—H1···N2^i^	0.88	2.25	2.9502 (16)	137
O1W—H1A···Cl1^ii^	0.94 (2)	2.18 (1)	3.1113 (13)	173 (2)
O1W—H1B···Cl1	0.95 (2)	2.28 (1)	3.2106 (12)	170 (2)
C5—H5···O1W^iii^	0.95	.	2.7203 (16)	156
C8—H8···Cl1^iv^	0.95	.	3.5862 (18)	137
N3—H3···Cl1^v^	0.88	2.94	3.7915 (15)	162
C2—H2···Cl1^v^	0.95	.	3.5604 (13)	159
C6—H6···Cl1^vi^	0.95	.	3.5329 (14)	127
C7—H7···Cl1^vi^	0.95	.	3.5649 (14)	123

Symmetry codes: (i) −*x*+1, −*y*, −*z*+1; (ii) −*x*+1, −*y*+1, −*z*; (iii) *x*, *y*−1, *z*; (iv) *x*+1, *y*, *z*; (v) −*x*+2, −*y*+1, −*z*+1; (vi) −*x*+2, −*y*, −*z*.
